# Supplementary material for: Causal Relationship between Aging and Anorexia Nervosa: A White-Matter-Microstructure-Mediated Mendelian Randomization Analysis
Source: Biomedicines. 2024 Aug 16;12(8):1874. doi: 10.3390/biomedicines12081874 (PMC11351342; doi:10.3390/biomedicines12081874)
Supplement: Supplementary file 1 [file biomedicines-12-01874-s001.zip › Supplementary Figure/Figure S8/Leaveoneout plot of MR analysis for TL to ubm-b-1933.pdf]

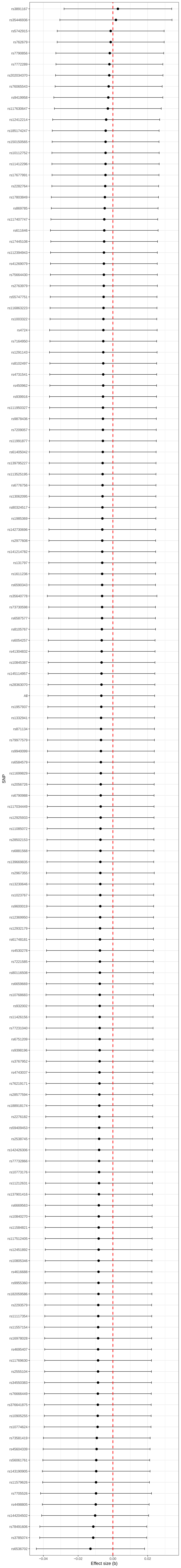
SNP
Effect size (b)
-0.04
-0.02
0.00
0.02
rs3891167
rs35446936
rs5742915
rs762679
rs7790856
rs7772289
rs202034370
rs76065543
rs9419958
rs117630647
rs12412214
rs185174247
rs150150565
rs10112752
rs11412296
rs17677991
rs2282764
rs17803849
rs869785
rs117407747
rs611646
rs17445108
rs112394943
rs41269079
rs75664430
rs2763979
rs55747751
rs116863223
rs1003322
rs4724
rs7164950
rs1291143
rs8102497
rs4731541
rs450962
rs939916
rs111950327
rs9878436
rs7209057
rs11991877
rs61405042
rs139795227
rs113525195
rs6776756
rs13062095
rs80324517
rs1985369
rs142730696
rs2977608
rs141214782
rs131797
rs1611236
rs6590343
rs35640778
rs73730598
rs6587577
rs8105767
rs6054257
rs41304832
rs10845387
rs145114957
rs28363070
All
rs1957937
rs1332941
rs871134
rs79977579
rs9940099
rs6584579
rs11699829
rs2056726
rs6790988
rs117034449
rs12925933
rs11085072
rs28502153
rs6881568
rs139669835
rs2967355
rs13230646
rs1023767
rs9600019
rs12369950
rs12932179
rs61748181
rs4530278
rs7221585
rs80116508
rs6659669
rs10768683
rs932002
rs11426156
rs77231040
rs6751209
rs9398196
rs3767952
rs4743037
rs76219171
rs28577594
rs188918174
rs2276182
rs59409453
rs2538745
rs142426306
rs77732866
rs10773176
rs11212631
rs137901416
rs6669563
rs10840270
rs11584821
rs117512405
rs12451892
rs10805346
rs4616688
rs9955360
rs182059586
rs2293579
rs11117354
rs11557154
rs16978028
rs4695407
rs11769630
rs2555104
rs34550383
rs76666449
rs376641875
rs10905255
rs10774624
rs73581419
rs45604339
rs56061761
rs143190905
rs11579626
rs7705526
rs4498805
rs144204502
rs78491606
rs3785074
rs6536702
